# Metabolic Glycoengineering Sensitizes Drug-Resistant Pancreatic Cancer Cells to Tyrosine Kinase Inhibitors Erlotinib and Gefitinib

**DOI:** 10.1016/j.bmcl.2015.01.060

**Published:** 2015-02-04

**Authors:** Mohit P. Mathew, Elaine Tan, Christopher T. Saeui, Patawut Bovonratwet, Lingshu Liu, Rahul Bhattacharya, Kevin J. Yarema

**Affiliations:** Department of Biomedical Engineering and the Translational Tissue Engineering Center, The Johns Hopkins University, Baltimore, Maryland, USA

## Abstract

Metastatic human pancreatic cancer cells (the SW1990 line) that are resistant to the EGFR-targeting tyrosine kinase inhibitor drugs (TKI) erlotinib and gefitinib were treated with 1,3,4-*O*-Bu_3_ManNAc, a “metabolic glycoengineering” drug candidate that increased sialylation by ∼12-fold. Consistent with genetic methods previously used to increase EGFR sialylation, this small molecule reduced EGF binding, EGFR transphosporylation, and downstream STAT activation. Significantly, co-treatment with both the sugar pharmacophore and the existing TKI drugs resulted in strong synergy, in essence re-sensitizing the SW1990 cells to these drugs. Finally, l,3,4-*O*-Bu_3_ManNAz, which is the azido-modified counterpart to l,3,4-*O*-Bu_3_ManNAc, provided a similar benefit thereby establishing a broad-based foundation to extend a “metabolic glycoengineering” approach to clinical applications.

## Graphical abstract

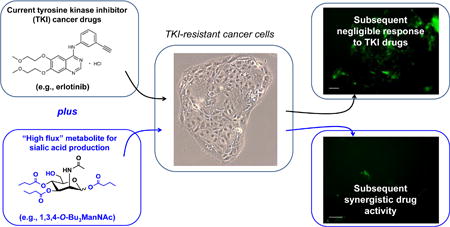

Tumor-associated carbohydrate antigens (TACAs) have been associated with cancer for decades^1,2^ and abnormal glycosylation is now accepted to be a universal feature of cancer.^3^ Despite the many roles glycosylation has been discovered to play in cancer progression and metastasis,^4-6^ progress in exploiting this knowledge in a clinical setting has been agonizingly slow.^7-8^ The epidermal growth factor receptor (EGFR) exemplifies the “sweet and sour”^8^ or “bittersweet”^7^ nature of glycosylation in cancer. On one hand, several papers over the past ∼15 years have reported that modulation of EGFR's glycosylation status – in particular changes to fucose and sialic acid^9^ – control this receptor's ability to drive cancer progression. For example, a recent report showed that increased sialylation or fucosylation of EGFR suppressed dimerization, inhibited subsequent phosphorylation, and dampened activation of downstream signaling in lung cancer cells.^9^ On the other hand, although these responses would be expected to slow cancer cell growth, the methods used to demonstrate these features in cell culture experiments, such as enzymatic removal of sugars or genetic over-expression of glycosyltransferases, are not easily translated to animal testing or to a human clinical setting thereby illustrating the general difficulties in developing carbohydrate-based cancer therapies.

In this report we overcome a hurdle towards clinical exploitation of the glycosylation status of EGFR by using small molecule “metabolic glycoengineering” sugar analogs to increase the sialylation of this receptor and reduce its activity in ways that were previously only accomplished genetically.^9,10^ Metabolic glycoengineering^11^ (also referred to as metabolic oligosaccharide engineering or MOE^12,13^) is a versatile technique used to change patterns of glycosylation by altering the availability or chemical composition of biosynthetic precursors of glycans.^14,15^ For example, short chain fatty acid (SCFA)-modified ManNAc analogs can efficiently enhance metabolic flux through the sialic acid biosynthetic pathway^16,17^ and increase cell surface sialylation.^18^ The ester-linked SCFA moieties, which are usually acetate^19-21^ or n-butyrate,^17,22^ increase cellular uptake by three orders of magnitude or more and upon entering a cell, intracellular esterases rapidly remove the SCFA groups,^23^ regenerating the core sugar that enters the targeted biosynthetic pathway. This strategy positions SCFA-modified sugars as viable drug candidates, as evidenced by the recent use of peracetylated ManNAc (Ac4ManNAc, shown in Figure 1) to reverse the symptoms of hereditary inclusion body myopathy in an animal model of this disease.^24^

One drawback of SCFA-conjugated monosaccharide drug candidates is cytotoxicity and growth inibition that, while not severe, can hinder metabolic incorporation into cell surface glycans.^16,17,25^ On the other hand, we have previously shown that cellular responses elicited by SCFA-modified ManNAc,^17,22,26^ which include down-regulation of NF-κB and metastatic oncogenes,^27^ have potential anti-cancer properties.^13,28^ Based on considerable evidence that O-acylated sialic acids can play roles in cancer biology,^29-33^ we were intrigued whether n-butyrate could be “carried through” the biosynthetic pathway and appear on the cell surface as O-butanyolated siaolosides. Careful mass spectrometry profiling of sialic acids (e.g., as reported in a recent publication^34^) failed to support this hypothesis however, Instead, our laboratory has uncovered structure activity relationships (SARs) that attribute analog-mediated cytotoxicity and high levels of growth inhibition to the presence of a SCFA group at the C6 position of a hexosamine.^28,35^ This discovery allowed us to develop tributanoylated sugars such as 1,3,4-*O*-Bu_3_ManNAc (Figure 1) that achieve high flux into the sialic acid pathway at low analog concentrations compared to the widely used peracylated compounds (e.g., Ac_4_anNAc).^18^ Importantly, use of the “1,3,4” analogs at concentrations less than 100 μM allow the side effects discussed above (e.g., cytotoxicity, severe growth inhibition, or NF-κB inhibition) to largely be avoided thus enabling a “glycan only” investigation of the impact of ManNAc analogs.

Of particular relevance to our efforts to develop glycosylation-based therapies for chemotherapy-resistant pancreatic cancer, we found that EGFR in SW1990 cells treated with l,3,4-*O*-Bu_3_ManNAc experienced an increase in sialylation of 2-fold or higher by using mass spectrometry-based “glycosite”^36^ and glycan analysis methods similar to those already reported^34,37^ (relevant experimental data is provided in the Supplemental Materials). Based on the precedent from at least two groups who used genetic methods to increase EGFR sialylation,^9,10^ we reasoned that l,3,4-*O*-Bu_3_ManNAc would likewise reduce EGFR activity. This hypothesis was experimentally confirmed by the experiments shown in Figure 2 where we first measured saturation binding to fluorescently-labeled EGF and showed a measurable decrease in available cell surface receptors for EGF (Figure 2A). In turn, immunofluorescence assays showed that analog treatment led to a decrease in EGFR phosphorylation (Figure 2B with additional images provided in Supplemental Figure SI), which was confirmed by western blots that also showed a decrease in phospho-EGFR (p-EGFR) in cells treated with l,3,4-*O*-Bu_3_ManNAc while the total amount of EGFR was unchanged (Figure 2C).

Although the changes in EGFR phosphorylation were relatively modest in l,3,4-*O*-Bu_3_ManNAc treated cells, small differences in starting conditions for receptors that initiate phosphorylation cascades typically are amplified downstream. To test whether this characteristic response of signaling pathways applied to l,3,4-*O*-Bu_3_ManNAc-driven changes to p-EGFR, phospho-STAT3 (p-STAT3) which is a “hallmark” downstream element of EGFR-initiated signaling, was monitored by western blot analysis. This experiment confirmed that the predicted enhanced response did occur for STAT3, with a stronger dose-dependent decrease in p-STAT3 levels observed in l,3,4-*O*-Bu_3_ManNAc-treated SW1990 cells as shown by western blots (Figure 3A) and quantification by Image J software (Figure 3B). The series of experiments beginning with reduction of EGF binding, progressing to show decreased EGFR phosphorylation, and culminating with dampened STAT activation brought about through the treatment of cells with l,3,4-*O*-Bu_3_ManNAc indicate that even modest changes in behavior of surface receptors due to altered glycosylation have potential therapeutic benefit. To gain a sense whether these biochemical markers of signaling pathway activation had an impact on actual cell behavior, we monitored cell proliferation and found that, given sufficiently high levels of l,3,4-*O*-Bu_3_ManNAc, SW1990 cell proliferation decreased (Figure 3C).

Despite the decreased cell proliferation observed in l,3,4-*O*-Bu_3_ManNAc treated cells, the high concentrations required to substantially inhibit growth (e.g., > 250 μM) may be difficult to achieve *in vivo*; therefore, the prospects for using l,3,4-*O*-Bu_3_ManNAc as a “stand alone” drug for inhibiting EGFR in TKI resistant cell lines are uncertain. The mechanism by which increased sialylation impacts EGFR, however – which is reported to be decreased dimerization and a concomitant reduction in transphosporylation^9^ – is orthogonal to other mechanisms by which cancer cells become resistant to TKIs such as single point mutations that affect the binding of these drugs to EGFR^38^ or the constitutive activation of ERK signaling that can circumvent EGFR inhibition.^39^ Therefore regardless of current uncertainties in the exact mechanism by which l,3,4-*O*-Bu_3_ManNAc modulates EGFR activation and signaling, we reasoned that co-administration of this drug candidate with TKIs such as erlotinib or gefitinib would have an synergistic effect and, in a best case scenario, re-sensitize drug-resistant cells

To evaluate synergy between l,3,4-*O*-Bu_3_ManNAc and TKIs, SW1990 cells were incubated with 0, 25, and 50 μM of l,3,4-*O*-Bu_3_ManNAc and then co-treated with a range of concentrations of erlotinib or gefitinib. These levels of 1,3,4-*O*-Bu_3_ManNAc are sufficient to increase sialylation^18^ and diminish phosphorylation of STAT (Figure 3B) but result in virtually no growth inhibition when the sugar analog is used alone (Figure 3C). As shown in Figure 4A neither erlotinib or gefinitib alone were effective at slowing proliferation SW1990 cells at low nanomolar concentrations but when used in combination with l,3,4-*O*-Bu_3_ManNAc the sensitivity to these drugs dramatically increased. Indeed, reduction in cell growth of 40 to 50% (which are levels associated with clinically effective response) occurred in the tens of nanomolar range for both erlotinib and gefitinib when the cells were co-treated with 50 μM of 1,3,4-*O*-Bu_3_ManNAc; this level of sensitivity to these TKIs are comparable to that observed in drug-responsive pancreatic cancer lines.^40^ In essence, the sugar analog reversed the drug resistance of this cell line.

The Combination Index (CI)^41^ was used to more rigorously quantify synergy between ManNAc analogs and existing drugs based on the following relationship:

$$\text{CI}=\frac{D_{1}}{{\left(D_{x}\right)}_{1}}+\frac{D_{2}}{{\left(D_{x}\right)}_{2}}$$

To calculate the CI, D_1_ (e.g., l,3,4-*O*-Bu_3_ManNAc) and D_2_ (e.g., erlotinib or gefitnib) represent the concentrations of each drug in combination with each other that required to inhibit cell growth by a given amount (x%) and (D_x_)_1_ and (D_x_)_2_ are the concentrations of each drug required to cause x% inhibition when the drugs are used individually. A CI = 1 indicates an additive effect, a CI > 1 indicates an antagonistic effect, and a CI < 1 indicates a synergistic effect.^41^ Figure 4B plots the CI measured for each analog-drug combination (using data from Figure 4A for the TKI drugs when used alone and Figure 3C for l,3,4-*O*-Bu_3_ManNAc alone). Strong synergy was observed between l,3,4-*O*-Bu_3_ManNAc and both erlotinib and gefitinib with combination indexes ranging from 0.7 to as low as 0.23. It is noteworthy that stronger synergy (e.g., lower CI values) was observed for 25 μM of l,3,4-*O*-Bu_3_ManNAc compared to 50 μM, indicating that the beneficial effects of this sugar analog “kick in” at low concentrations. Overall, these results position sugar analogs such as 1,3,4-*O*-Bu_3_ManNAc as valuable agents for sensitizing drug-resistant pancreatic cancer to existing therapies that target EGFR.

A final set of experiments investigated whether the azide-modified ManNAc analog 1,3,4-*O*-Bu_3_ManNAz (shown in Figure 1), which is incorporated into cell surface glycans as the non-natural “Sia5Az” form of sialic acid,^42^ also provides synergy with EGFR-targeting TKI drugs. The use of a sialic acid precursor with a non-natural acyl group was of interest for two reasons; one was based on a previous report that this class of compounds could sensitize cancer cells towards chemotherapy and radiation treatment.^43^ Secondly, azido-modified sugar analogs are capable of labeling cells through click chemistry,^18,42^ thereby opening new possibilities of not only synergistically combining carbohydrate analogs with EGFR-acting drugs, but to also provide a means to direct additional diagnostic or therapeutic agents to sialylated glycans overexpressed in cancer.^44,45^ In the current experiments, azido-modified l,3,4-*O*-Bu_3_ManNAz was more cytotoxic on its own (Figure 4C) compared to l,3,4-*O*-Bu_3_ManNAc, therefore lower concentrations of 12.5 and 25 (μM were used to evaluate synergy (Figure 4D). Even at these low levels of l,3,4-*O*-Bu_3_ManNAz, strong incorporation into cellular glycans occurs^18^ and synergy with erlotinib was observed with CI values ranging from 0.68 to as low as 0.34 (Figure 4E).

In summary, this brief report describes two important findings, the first is that the manipulation of EGFR glycosylation in ways that reduce signaling and dampen endpoints related to cancer progression can now be done with pharmacologically-relevant small molecules rather than the genetic approaches previously reported. The second finding is that treatment of TKI-resistant pancreatic cancer cells with the sugar analogs can restore sensitivity to erlotinib and gefitinib. These results provide a scientific foundation for further investigation beyond the scope of this report; for example it is not known how widely the drug synergy will apply across cell lines and tissue types and a detailed accounting of the mechanism by which synergy is achieved remains to be described.

## Supplementary Material

1

2

## Figures and Tables

**Figure 1:**
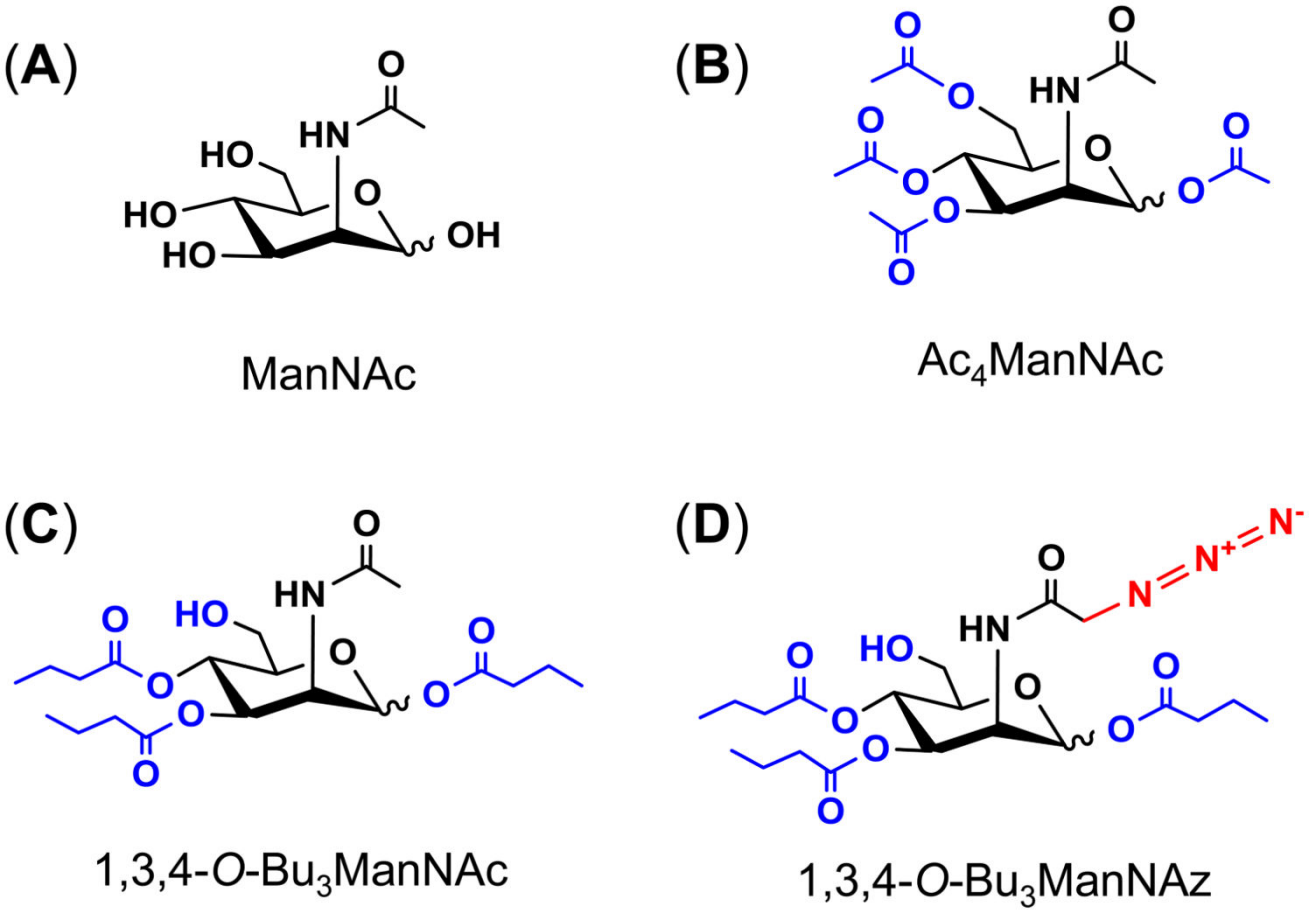
ManNAc and analogs used for metabolic glycoengineering pancreatic cancer cells for increased sensitivity to EGFR-targeting TKI drugs (**A**) Natural ManNAc. (**B**) Fully acetylated ManNAc, Ac_4_ManNAc. (C) “High-flux” tributanoylated ManNAc, l,3,4-*O*-Bu_3_ManNAc. (D) Azide-modified high flux ManNAc, l,3,4-*O*-Bu_3_ManNAz.

**Figure 2:**
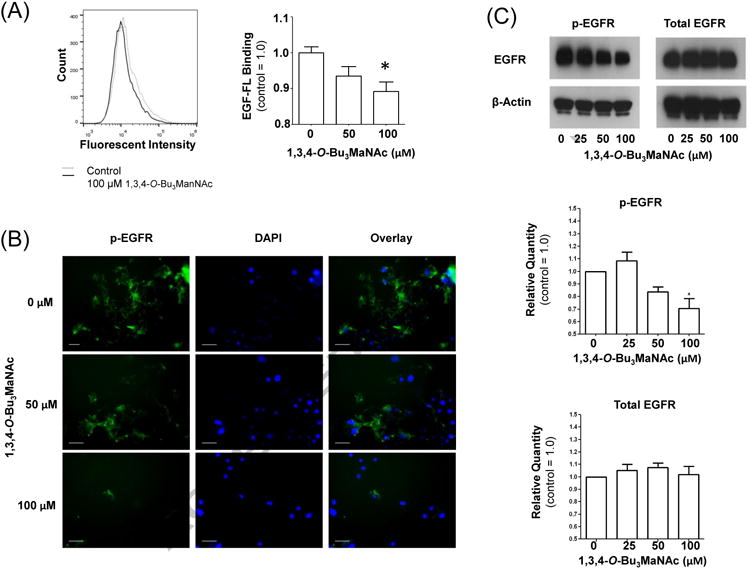
l,3,4-*O*-Bu_3_ManNAc decreases EGFR phosphorylation (**A**) Saturation binding where cells were incubated with Alexa Fluor® 488 labeled EGF for one hour at room temperature and then measured using flow cytometry shows a decrease in available surface bound EGFR. At least 3 biological replicates were carried out for each experiment with data expressed as mean ± standard error mean (SEM). (**B**) Representative images of immunofluorescence assays (additional images are provided in Supplemental Figure SI) where cells were incubated with EGF for 2.0 min, fixed and stained with anti-p-EGFR, FTTC labeled anti-rabbit antibody, and stained with DAPI confirm that EGFR phosphorylation decreases with analog treatment. (**C**) Western blots of SW1990 pancreatic cancer cells treated with increasing levels of l,3,4-*O*-Bu_3_ManNAc showed a decrease in phosphorylated EGFR with no significant change in overall EGFR levels. * indicates a p value of < 0.05.

**Figure 3:**
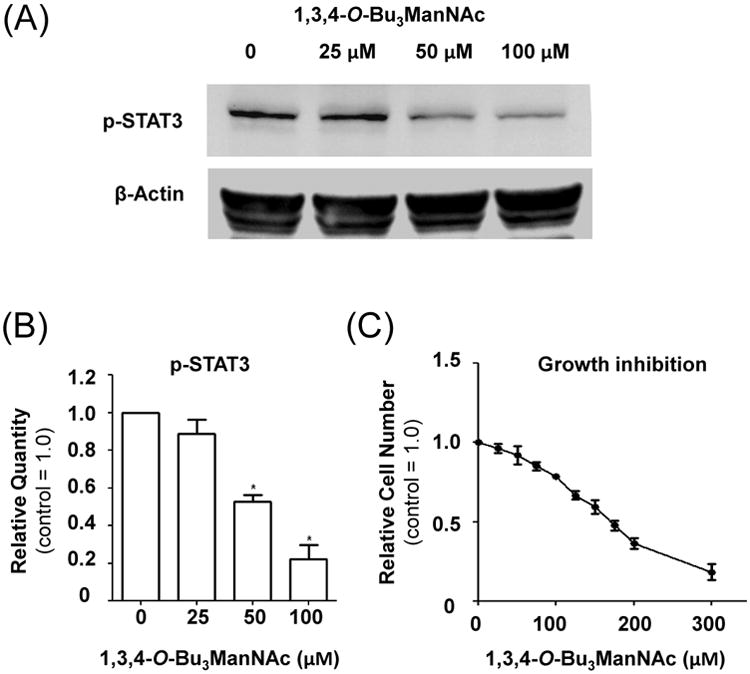
Downstream effects of l,3,4-*O*-Bu_3_ManNAc treatment of SW1990 cells and reduced EGFR phosphorylation (**A**) Representative images of western blots of SW1990 cells treated with different concentrations of l,3,4-*O*-Bu_3_ManNAc showed a significant decrease in phosphorylated STAT3 (as quantified by Image J analysis in Panel **B**). (**C**) Finally, l,3,4-*O*-Bu_3_ManNAc reduced cell proliferation albeit at higher concentrations than where effects on EGFR-mediated signaling endpoints were observed. At least 3 biological replicates were carried out for each experiment with data expressed as mean + standard error mean (SEM). * indicates a p value of < 0.05.

**Figure 4:**
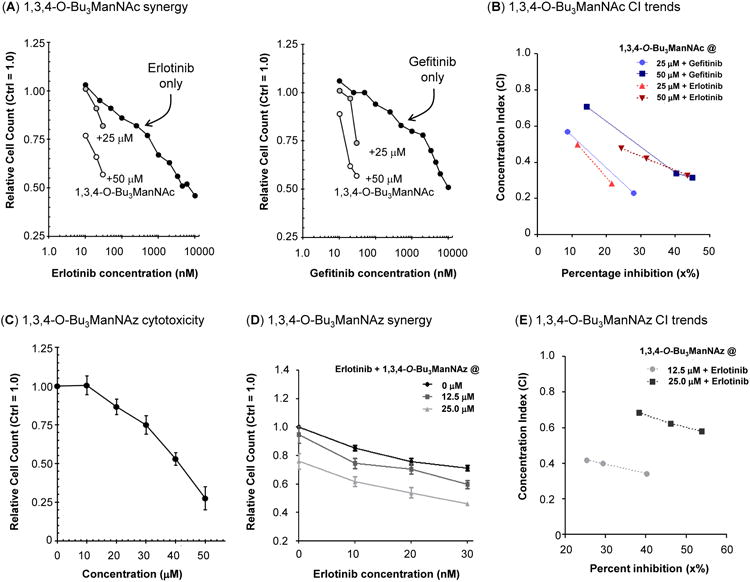
Determination of synergy between glycosylation and EGFR-acting drugs (**A**) Growth inhibition of SW1990 cells treated with gefitinib or erlotonib in combination with l,3,4-*O*-Bu_3_ManNAc. (**B**) The Combination Index (CI) for each analog-drug combination was determined and plotted against the percentage inhibition observed (x%), with both drugs showing strong synergy with the sugar analog. At least 3 biological replicates were carried out for each experiment with data expressed as mean ± standard error mean (SEM) as depicted in Supplemental Figure S2. (**C**) Growth inhibition of SW1990 cells treated with l,3,4-*O*-Bu_3_ManNAz. (**D**) Growth inhibition when erlotonib and l,3,4-*O*-Bu_3_ManNAz were used in combination with the combination index provided in Panel (**E**). At least 3 biological replicates were carried out for each experiment with data expressed as mean ± standard error mean (SEM).
