# Supplementary material for: Metabolic Glycoengineering Sensitizes Drug-Resistant Pancreatic Cancer Cells to Tyrosine Kinase Inhibitors Erlotinib and Gefitinib
Source: Bioorg Med Chem Lett. Author manuscript; Available in PMC 2018 Jan 4. (PMC5753412; doi:10.1016/j.bmcl.2015.01.060)
Supplement: 1 [file NIHMS661164-supplement-1.docx]

**Supplemental Materials for**

*Metabolic Glycoengineering Sensitizes Drug-Resistant Pancreatic Cancer Cells to Tyrosine Kinase Inhibitors Erlotinib and Gefitinib*

*Mohit P. Mathew^1^, Elaine Tan^1^, Christopher T. Saeui, Patawut Bovonratwet, Lingshu Liu, Rahul Bhattacharya, and Kevin J. Yarema^2^*

Department of Biomedical Engineering and the Translational Tissue Engineering Center

The Johns Hopkins University, Baltimore, Maryland, USA

***This document contains***:

**Supplemental Figure 1** (on Page 2): This figure shows additional “representative data” from immunofluorescence assays that demonstrate the ability of 1,3,4-*O*-Bu_3_ManNAc to reduce p-EGFR levels in SW1990 cells. Similar data is provided in Figure 2 of the parent publication.

**Supplemental Figure 2** (on Page 3): This figure provides a more detailed depiction of low concentration ranges of the experiment used to determine drug synergy between 1,3,4-*O*-Bu_3_ManNAc and erlotinib and gefitinib in **Figure 4A** of the main text.

**Experimental Methods** (on Page 4 and following): A detailed description of the experimental methods that are beyond the scope of the main text are provided.

**Glycoproteomics Data** (provided as a separate Excel file titled “Glycoproteomics Data”). This data was obtained following the glycosite analysis procedures described previously for SW1990 cells treated with 1,3,4-O-Bu_3_ManNAc [[1](#_ENREF_1)]. The key piece of data relevant to this study is provided on line 216, column AQ where the ratio of sialylated EGFR in analog-treated compared to untreated control cells is provided.

**Supplemental Figure 1**. Representative images at 10 X magnification of immunofluorescence assays where cells were incubated with EGF for 2.0 min, fixed and stained with anti-p-EGFR, FITC labeled anti-rabbit antibody, and DAPI confirm that EGFR phosphorylation decreased with sugar analog treatment. The scale bar represents 100 μm.

**
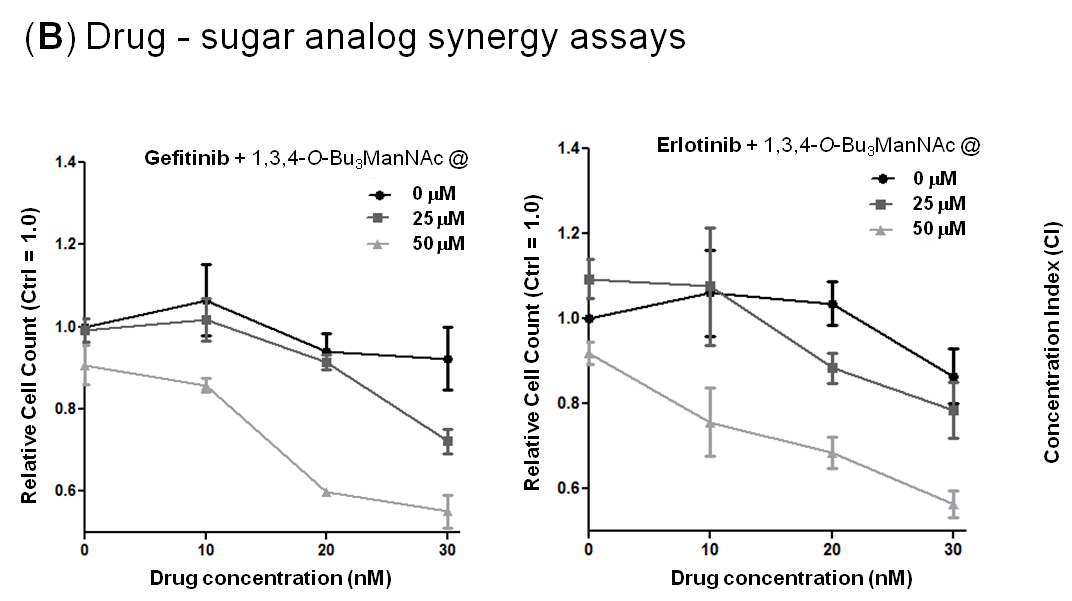
**

**Supplemental Figure 2**. Details showing additional data obtained over low concentration ranges for the drug synergy experiments presented in Figure 4A in the main text. At least 3 biological replicates were carried out for each experiment with data expressed as mean ± standard error mean (SEM)

**EXPERIMENTAL METHODS**

**Cell Culture and Incubation with Sugar Analogs**

# SW1990 (ATCC^®^ CRL-2172) cells were grown in Dulbecco's Modified Eagle Medium (DMEM) supplemented 10 % with heat-inactivated fetal bovine serum (FBS) and 1.0 % of 100x pen/strep antibiotic solution (Invitrogen). Cells were maintained at 37 °C in a humidified atmosphere containing 5% CO_2_. For treatment of cells, cells typically were plated in 6-well tissue culture plates in 2.0 mL of culture media at a density of 300,000 cells/well. Sugar analogs, either 1,3,4-*O*-Bu_3_ManNAc or 1,3,4-*O*-Bu_3_ManNAz were synthesized and characterized as previously described [[2](#_ENREF_2), [3](#_ENREF_3)] and stored lyophilized at -80 °C. Stock solutions (100 mM) were made in ethanol (EtOH) and the compounds were added to each well to achieve the desired analog concentrations; the identical volume of ETOH (always less than 10 μL/mL) was added to each well in each experiment to ensure that all cells were exposed to the same amount of solvent as those treated with the highest concentration of analog. Cells were typically incubated for 48 h with the sugar analogs; in many experiments (as indicated below) the first 24 h of incubation was carried out in complete media and the cells were serum starved for the final 24 h before analysis following published protocols for monitoring EGFR phosphorylation and activation [[4](#_ENREF_4)].

**EGF Saturation Binding Assays**

Cells were incubated for 48 h with 1,3,4-*O*-Bu_3_ManNAc with serum starvation over the last 24 h. The cells were washed with PBS, treated with enzyme free cell dissociation buffer (Life Technologies) until they detached from the culture plate, collected, and counted and cell numbers were normalized using the Beckman Z2 cell coulter counter. Cells were then washed twice in Live Cell Imaging Solution (Life Technologies) supplemented with 1.0% bovine serum albumin (BSA) and 20 mM glucose. Cells were then incubated at room temperature for 2 h with 2 µg/mL of Alexa Fluor 488-linked EGF (Life Technologies). Cells were washed three times in Live Cell Imaging Solution and analyzed using flowcytometry with an Accuri C6 Flow cytometer.

# Immunofluorescence

Cells were analyzed by fluorescence microscopy after incubation with analogs for 48 h including serum-starvation over the last 24 h after which they washed with phosphate buffered saline (PBS) and then incubated with 10 ng/mL recombinant human EGF (Peprotech AF-100-15) in PBS for 2.0 min to activate EGFR signaling from basal levels [[4](#_ENREF_4)]. Cells were then fixed, permeabilized, blocked and incubated with human specific anti-phospho-EGFR (Cell Signaling) primary antibody for 1.0 h at room temperature. After incubation with the primary antibody, cells were washed and incubated with FITC-linked anti-rabbit (Sigma-Aldrich) secondary antibody for 1.0 h at room temperature. Cells were then washed and incubated with 4,6-diamidino-2-phenylindole (DAPI) for 10 min to stain the nuclei of the cells. After incubation, cells were washed and images were taken using an excitation wavelength of 495 nm and an emission wavelength of 519 nm with a Zeiss Observer A1 microscope.

**Western Blot Analysis**

Proteins obtained from SW1990 cells were analyzed by western blots after the cells were incubated with 1,3,4-*O*-Bu_3_ManNAc for 48 h including, as described above, serum starvation for the last 24 h and exposure to 10 ng/mL recombinant human EGF (Peprotech AF-100-15) in PBS for 2.0 min. Proteins were collected and were immuno-detected using the following commercial antibodies: anti-phospho-EGFR (Cell Signaling), anti-EGFR (Cell Signaling), anti-phospho-STAT3 (Cell Signaling), anti-β-actin (Sigma-Aldrich) and HRP-linked anti-rabbit antibody (Cell Signaling). Protein bands were quantified using the ImageJ software.

# Growth Inhibition Assays to Determine Drug Synergy

# Cells were incubated with 1,3,4-*O*-Bu_3_ManNAc or 1,3,4,-*O*-Bu_3_ManNAz for 48 h, typically at low concentrations (e.g., 25 or 50 μM) to avoid sugar analog toxicity, after which the media was changed and incubation was continued in the presence of the sugar analog but with gefitinib or erlotinib (Sigma-Aldrich) added to the wells. After an additional 72 h incubation period the cells were washed, trypsinized, and counted using a Beckman Z2 cell coulter counter.

**Statistical Analysis**

Data was expressed as means ± standard error (SEM). Statistical significance was determined using one way ANOVA with a dunnett’s post-test to compare means of different samples with the control. The null hypothesis was rejected in cases where p-values were < 0.05.

**References**

1 Almaraz, R. T., Tian, Y., Bhattarcharya, R., Tan, E., Chen, S.-H., Dallas, M. R., Chen, L., Zhang, Z., Zhang, H., Konstantopoulos, K. and Yarema, K. J. (2012) Metabolic flux increases glycoprotein sialylation: implications for cell adhesion and cancer metastasis. Mol Cell Proteomics, 10.1074/mcp.M1112.017558

2 Aich, U., Campbell, C. T., Elmouelhi, N., Weier, C. A., Sampathkumar, S. G., Choi, S. S. and Yarema, K. J. (2008) Regioisomeric SCFA attachment to hexosamines separates metabolic flux from cytotoxicity and MUC1 suppression. ACS Chem Biol. **3**, 230-240

3 Almaraz, R. T., Aich, U., Khanna, H. S., Tan, E., Bhattacharya, R., Shah, S. and Yarema, K. J. (2012) Metabolic oligosaccharide engineering with *N*-acyl functionalized ManNAc analogues: cytotoxicity, metabolic flux, and glycan-display considerations. Biotechnol Bioeng. **109**, 992-1006

4 Liu, Y. C., Yen, H. Y., Chen, C. Y., Chen, C. H., Cheng, P. F., Juan, Y. H., Chen, C. H., Khoo, K. H., Yu, C. J., Yang, P. C., Hsu, T. L. and Wong, C. H. (2011) Sialylation and fucosylation of epidermal growth factor receptor suppress its dimerization and activation in lung cancer cells. Proc Natl Acad Sci U S A. **108**, 11332-11337
